# Cascade Electrocatalytic Conversion of CO_2_ to C_3_ Products at Elevated Pressures

**DOI:** 10.1002/cssc.202500695

**Published:** 2025-06-10

**Authors:** Nandalal Girichandran, Lakshmi Mohan, Sanne Buisman, Andrew Morrison, Ruud Kortlever

**Affiliations:** ^1^ Process & Energy Department, Faculty of Mechanical Engineering Delft University of Technology Leeghwaterstraat 39 2628 CB Delft Netherlands; ^2^ Electrochemical Innovation Lab, Department of Chemical Engineering University College London London WC1E 7JE UK; ^3^ The Faraday Institution, Quad One, Harwell Science and Innovation Campus Didcot OX11 0RA UK

**Keywords:** 2‐propanol, cascades, electrocatalysis, electrochemical CO_2_ reductions, elevated pressures

## Abstract

Recent progress in the electrochemical reduction of CO_2_ (CO_2_RR) has led to notable breakthroughs in generating C_2_ compounds such as ethylene and ethanol. Nevertheless, the direct formation of C_3_ products encounters significant limitations due to the C_2_–C_1_ coupling reaction, posing a considerable challenge to improving their faradaic efficiency. Here, a design for an elevated pressure cascade catalytic reactor to convert CO_2_ to C_3_ products in a two‐step electrochemical process is presented. At 25 bar pressure, by regulating the potential of the cascade system and the electrolyte flow rate, a 40% selectivity for 2‐propanol on a copper electrode placed upstream of a silver electrode that converts CO_2_ to CO is reported. In cascade mode (with both silver and copper electrodes active), the C_3_:C_2_ oxygenate ratio significantly increases to 7 compared to the noncascade mode (copper only) with a modest ratio of about 0.6. Therefore, our elevated pressure cascade electrolysis approach demonstrates a notable step forward in CO_2_ electroreduction to oxygenated C_3_ products.

## Graphical abstract

By applying elevated CO_2_ pressure to a sequential cascade electrocatalytic system, composed of an upstream silver and downstream copper electrode, unique C‐C coupling pathways towards higher alcohols such as 2‐propanol are enhanced. At 25 bar, an unprecedented 2‐propanol selectivity of 40% by tuning the potential of the cascade system and flow rate of the electrolyte is reported.

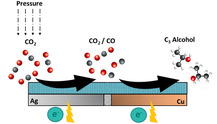

## 1 Introduction

The electrochemical CO_2_ reduction reaction (CO_2_RR) is a promising approach to electrochemically convert waste carbon dioxide into value added products. The CO_2_RR offers a promising pathway for reducing carbon emissions while simultaneously producing useful chemicals or fuels, contributing to a more sustainable and environmentally friendly approach to energy conversion.^[^ ^1^ ^]^ Electrocatalysts play a crucial role in facilitating the CO_2_RR, by lowering the energy barrier for CO_2_ conversion and determining the obtained product selectivity.^[^ ^2^, ^3^ ^]^ Significant research efforts have gone into developing improved catalytic materials by altering their facets, structure, or alloying.^[^ ^4^, ^5^, ^6^ ^]^ One of the primary focuses in CO_2_RR research has been the development of suitable catalytic systems to selectively produce multicarbon products such as ethylene, ethanol, and propanol. However, the production of these multicarbon products on a single catalyst is limited by scaling relations that prevent the optimization of key steps in the overall reaction mechanism.^[^ ^7^, ^8^ ^]^

To address these catalytic limitations, recent research has shifted towards combining catalyst pairs that work synergistically, either in a tandem manner (no spatial separation) or cascade manner (spatial separation).^[^ ^9^, ^10^ ^]^ Cascade catalysis is a multistep process involving multiple catalysts where a reactant is first converted to an intermediate product on one catalyst that gets consumed on another catalyst to yield the desired product. Cascading has been demonstrated as an interesting alternative approach for the CO_2_RR to produce products that arise from CO as a primary intermediate.^[^ ^11^, ^12^, ^13^ ^]^ Theaker et al. used silver and copper electrodes in two separate reactors to produce ethanol from CO_2_ via CO as the intermediate.^[^ ^12^ ^]^ The conversion of CO was poor (<10%) due to CO being diluted between the reactors owing to mixing with other components and its poor solubility in the aqueous electrolyte. In a study using gold and copper interdigitated cascaded electrodes within a single reactor, Lum et al. showed that CO formed on gold spills over via diffusion to copper thereby increasing its concentration well beyond its solubility limit.^[^ ^14^ ^]^ This resulted in an enhanced synthesis of C—C coupled products from CO_2_. Interestingly, the obtained amounts of oxygenates (ethanol) was higher than that of hydrocarbons (ethylene), which is consistent with a recent study on high‐pressure CO reduction on copper that highlights the importance of achieving a high CO coverage to obtain higher reduction liquid products.^[^ ^15^ ^]^ In a different interpretation of the same concept using their custom setup, Gurudayal et al. used convection to aid the transport of CO formed on a silver electrode to a downstream copper electrode.^[^ ^10^ ^]^ By controlling the flow rate and applied potential, an optimal ratio of oxygenates to hydrocarbons was achieved. Yet, the most prominently observed products were restricted to the commonly reported ethanol, with small amounts of acetate and acetaldehyde. The formation of C_3_ products such as propanol remains challenging, pointing to suboptimal reaction conditions.

Tandem and cascade catalyst systems involving Cu and Ag have been identified as promising configurations for enhancing CO_2_ electroreduction via a cascade mechanism, where CO generated on Ag can be efficiently utilized on adjacent Cu sites for further reduction to multicarbon products.^[^ ^16^ ^]^ Such spatial arrangements are critical, as they promote effective intermediate transfer, maintain high local CO concentrations at the Cu surface, and thereby enable more favorable conditions for C—C coupling. This design principle reinforces the relevance of spatially structured cascade systems for accessing higher‐order products beyond ethanol and ethylene, particularly under conditions that optimize intermediate availability and surface reactivity.

Theoretical studies predict that the coverage of the key intermediates *CO and *H on a copper surface, and therefore the product distribution, is contingent upon factors such as surface morphology, applied potential, and importantly, the applied pressure.^[^ ^15^, ^17^, ^18^, ^19^, ^20^, ^21^ ^]^ The main benefits of pressurization are that it negates the solubility limitations of CO_2_ and CO in aqueous media and thereby increases the surface coverage of the reactants and intermediates on copper, resulting in enhanced production of higher carbon products such as alcohols, and aldehydes.^[^ ^15^ ^]^ Higher surface coverages of *CO on the surface of copper can significantly decrease the binding energies for *CO and other key intermediate species, paving the way towards uncommon products.^[^ ^15^, ^20^ ^]^ For instance, in a recent study using an electrolyte supersaturated with CO_2_, Kun et al. reported an enhanced production of 2‐propanol/isopropanol (IPA) with a selectivity of 56% on a silver‐copper alloy catalyst prepared under similar supersaturated conditions.^[^ ^22^ ^]^ Moreover, in our own previous work using elevated pressure for CO_2_RR on a copper foam electrode, we achieved a 11% selectivity towards 2‐propanol.^[^ ^23^ ^]^ The low amounts of C_3_ products observed during atmospheric pressure cascade CO_2_ electroreduction could be an indication of poor surface coverages of *CO and other reactant species.^[^ ^21^ ^]^ This hypothesis is further supported by the prediction of Sandberg et al. that the *CO and *H coverage impacts the surface dimerization and hydrogenation rate.^[^ ^20^ ^]^

We posit that applying elevated CO_2_ pressure to a cascade catalytic system composed of an upstream silver and downstream copper electrode can enable and enhance unique C—C coupled pathways towards elusive higher alcohols like 2‐propanol. To test this hypothesis, we have designed a novel high pressure electrochemical reactor for cascade CO_2_ reduction. Using this setup, a simplified schematic is provided in section S2, Supporting Information, we deliver a sufficiently concentrated supply of the intermediate CO to the copper electrode. Strikingly, at a pressure of 25 bar, we find a 2‐propanol selectivity of 40% on a copper foil electrode by tuning the potential of the cascade system and flow rate of the electrolyte. Moreover, the C_3_:C_2_ oxygenates ratio, mainly 2‐propanol (C_3_) and ethanol, acetaldehyde, and ethylene glycol (C_2_), increases to 7 for cascade operation mode (with both silver and copper electrodes active) compared to a mere 0.62 for the noncascade operation mode with only Cu as active electrode.

## 2 Results and Discussion

### 2.1 Electrochemical Cell design

**Figure 1:**
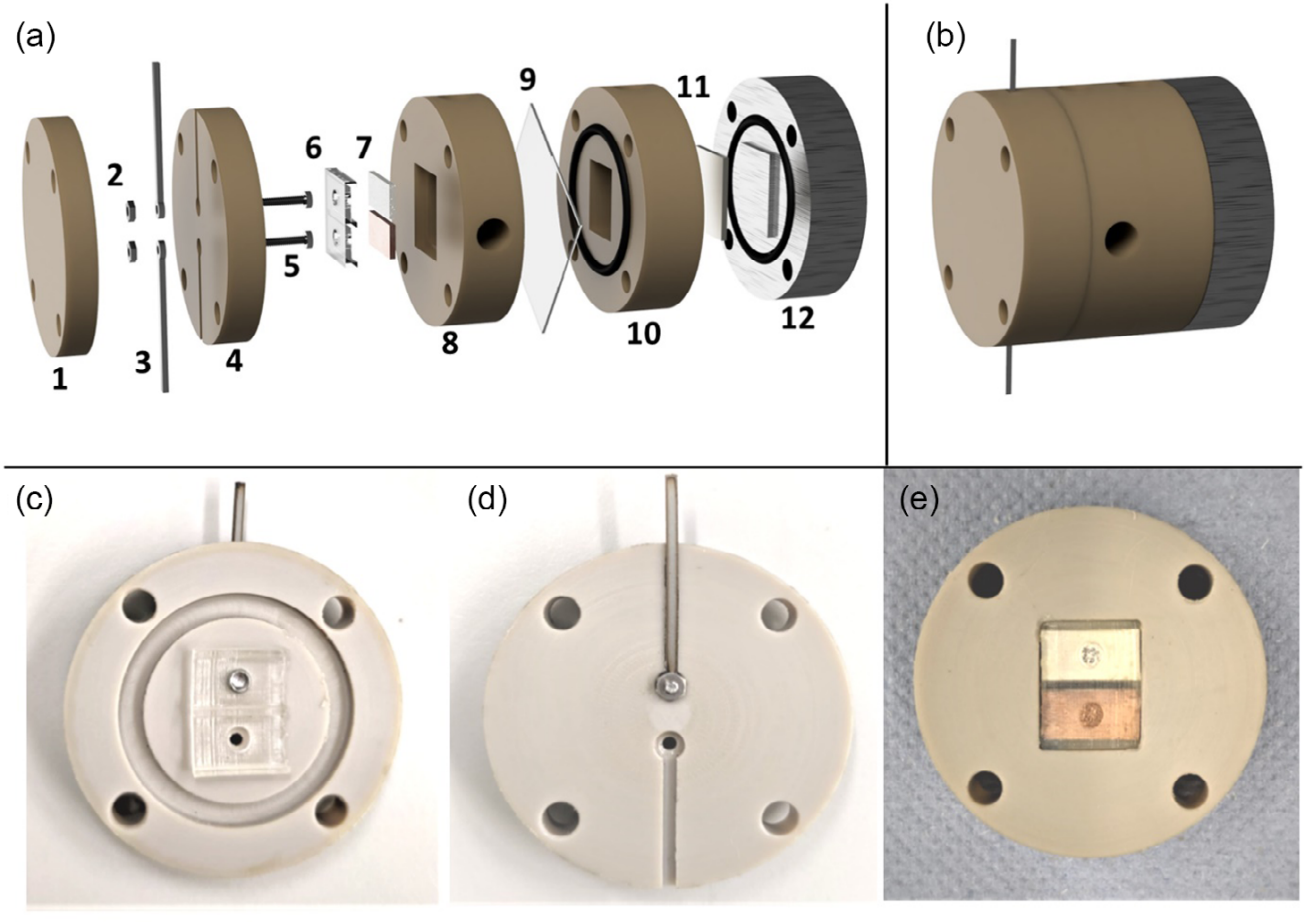
Custom‐designed high‐pressure electrochemical cell for cascade CO_2_ reduction. a) Exploded view showing major components including endplates, electrode holder, and flow chambers—(1) endplate cathode back, (2) connection nuts, (3) connection rods, (4) endplate cathode front, (5) connection bolts, (6) electrode holder (made from Flexible 80 A resin, Formlabs), (7) cathodes, (8) catholyte chamber, (9) membrane, (10) anolyte chamber, (11) anode, (12) conductive endplate. b) Closed electrochemical cell. c) Front view of cathode endplate with electrode holder in place. d) Back view showing electrical connection ports. e) Catholyte chamber with mounted Ag and Cu electrodes. The design enables spatial separation and independent control of each electrode in bipotentiostatic mode.

To implement this cascade concept under elevated pressure, we constructed a custom electrochemical cell as shown in **Figure** **1**. A detailed discussion of the high‐pressure device supporting the electrochemical cell—including its mechanical integrity, sealing strategy, design choices, and safe operation under elevated pressures—is provided in our previous study.^[^ ^24^ ^]^ The cell comprises a stainless‐steel anode end plate (grade: 304/1.4301, Xometry Europe GmbH) on one side and a PEEK cathode end plate on the other side, sandwiching the catholyte and anolyte flow plates that are separated by a Nafion 117 membrane. At the anode side, the electrical connection is realized by the steel endplate, connected to the electrode. Since the anode (Figure 1a—part 11) is located inside the slot in the flow plate, a protrusion from the steel end plate pushes the electrode into the slot thereby ensuring a good electrical connection. At the cathode side, a 3D‐printed (Elastic 50 A resin, Formlabs) frame (Figure 1c) was designed (with similar dimensions as the electrode slot in the flow channel (Figure 1e) to secure both the working electrodes in place, while offering openings at the back to provide separate electrical contacts. The material of the holder is insulating and a protrusion between the electrodes ensures they are insulated from each other, a criterion crucial to operate them in a bipotentiostatic mode. This material was selected for the holder as it can bend, stretch, compress, and withstand repeated use without tearing or quickly springing back to its original shape. This ensures that post assembly and during operation at high pressures, the holder can stretch and seal the chamber, making it leak tight.

Two cutouts at the back of the holder allow electrical connections (Figure 1d). Custom laser cut steel rods were used to provide connection between the external potentiostat and the electrodes. A bolt provided electrical contact between the back of the electrodes and the steel rods. To ensure there will be a connection between the bolt and the rod, a nut was added. This nut was used to secure the rod into the groove in the back of the plate, using the thread of the bolt (Figure 1a—parts 2, 3, and 5). Grooves in the endplate conceal the nuts, allowing a seamless stacking of plates. A small piece of aluminum tape (pressed into a circular shape of diameter ≈3 mm) was added to the head of the bolts before assembly to guarantee consistent contact with electrodes in the holder. The 3D‐printed electrode holder was designed with adaptability in mind. Opting for a holder, as opposed to relying solely on pressing the electrodes into the flow plate, offers the notable advantage that, when modifying operational configurations and conditions, only this element necessitates replacement sparing the need for a complete overhaul of the entire reaction chamber. This way it becomes possible to vary the distance between the electrodes, the electrode sizes, or their configuration with ease.

### 2.2 Electrochemical CO_2_ Reduction at High Pressure

**Figure 2:**
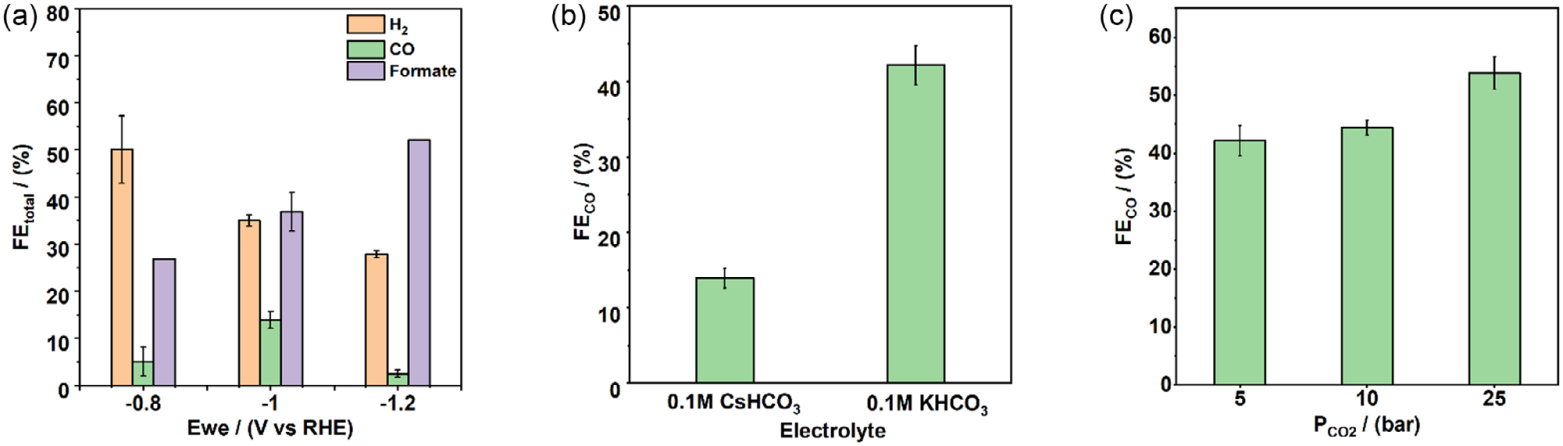
CO production performance of Ag electrode under varying electrochemical conditions. a) FE of products as a function of applied potential at 5 bar in 0.1 M CsHCO_3_ (25 mL min^−1^). b) Comparison of FE_CO_ using CsHCO_3_ and KHCO_3_ electrolytes at −1.0 V vs RHE. c) FE_CO_ at −1.0 V vs RHE as a function of applied pressure in 0.1 M KHCO_3_. Error bars represent standard deviation from at least two independent measurements.

The electrochemical performance of the cascade system was studied using chronoamperometry (CA) to investigate the impact of potential towards product selectivity. As a first step, a CO_2_ pressure of 5 bar was applied, and the potential on the Ag electrode was altered to determine the optimal conditions for the supply of CO to the Cu electrode, the second part of the cascade. For all the measurements discussed in the following, the electrolyte was circulated at 25 mL min^−1^ through the cell unless explicitly mentioned otherwise. The initial tests were conducted with 0.1 M CsHCO_3_ to compare this study to the pioneering work of Lum et al.^[^ ^14^ ^]^ and Gurudayal et al.^[^ ^10^ ^]^ on this topic. Three different potentials (−0.8, −1, and −1.2 V vs RHE) were applied on the Ag electrode while keeping copper inactive. The major products produced were H_2_, CO, and formate (**Figure** **2**a). The faradaic efficiency (FE) of H_2_ decreased from 50 to 28% with an increase in potential, while formate production displayed the opposite trend with a FE starting at 27% at −0.8 V vs RHE and reaching 52% at −1.2 V vs RHE. For CO, the FE followed a volcano‐like trend peaking at 14% at −1 V vs RHE. We therefore chose to apply −1 V vs RHE on silver for all further cascade experiments as this was the potential at which the highest amount of CO was obtained. Studies on the microenvironment near an Ag electrode have revealed that larger cations with smaller hydration shell such as Cs^+^ have a stronger electrostatic interaction with the electrode surface than K^+^. This results in a stronger stabilization of reaction intermediates such as *CO and *COOH with strong dipole moments.^[^ ^25^, ^26^ ^]^ This coupled with the stronger buffering strength^[^ ^27^ ^]^ of Cs^+^ compared to K^+^ can result in a slightly lower pH leading to preferential formation of HCOOH over CO while using CsHCO_3_ (alkaline pH favors CO formation on silver).^[^ ^25^ ^]^ Based on this understanding, CO_2_RR experiments were also conducted at 5 bar by applying −1 V vs RHE on Ag using 0.1 M KHCO_3_ (Figure 2b). We observed that the FE_CO_ increased to 42% for K^+^ instead of 14% for Cs^+^. Since the goal is to maximize the supply/amount of CO delivered to the downstream Cu electrode for further reaction, 0.1 M KHCO_3_ was used as the electrolyte for the rest of the experiments. Figure 2c shows the effect of a pressure increase on the FE_CO_ using 0.1 M KHCO_3_ on Ag catalyst. At 10 and 25 bar, the FE_CO_ increases further to 44% and 54%, respectively, indicating that the higher applied pressure^[^ ^28^ ^]^ (in this study limited to 25 bar) is better suited to supply higher amounts of CO to the downstream Cu electrode.

**Figure 3:**
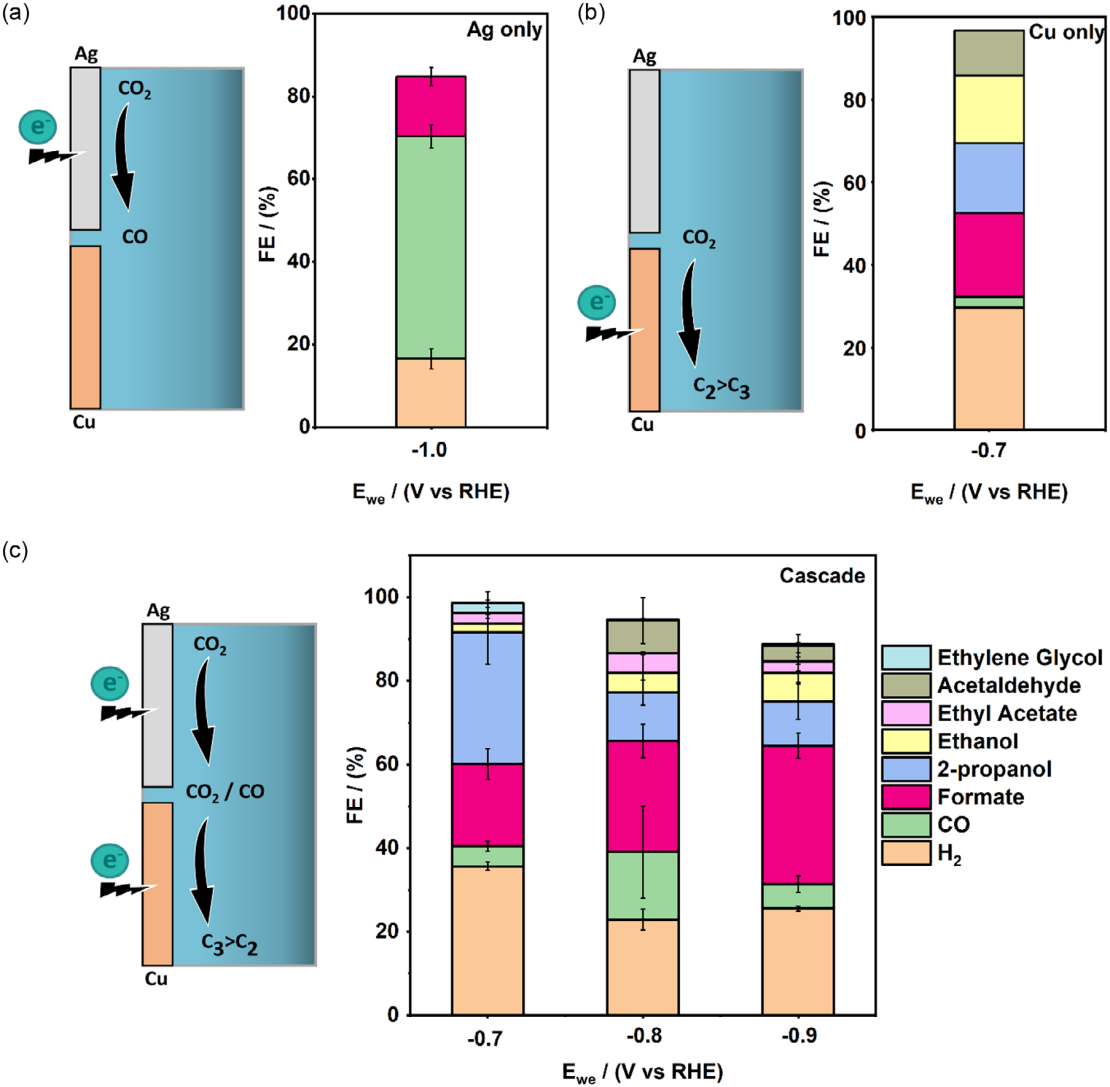
Faradaic efficiencies for key products under different electrode configurations. a) Ag‐only operation (Cu inactive) at multiple potentials. b) Cu‐only operation (Ag inactive) at −0.7 V vs RHE. c) Cascade mode with Ag at −1.0 V and Cu varied from −0.7 to −0.9 V vs RHE. Electrolyte: 0.1 M KHCO_3_, recirculated at 25 mL min^−1^, 25 bar. Error bars represent standard deviation from at least two independent measurements.

Cascade experiments were successfully conducted at 25 bar by fixing the Ag electrode at −1 V vs RHE while varying the potential on an oxide derived Cu electrode (−0.7, −0.8, and −0.9 V vs RHE). The product distribution in cascade mode is shown in **Figure** **3**c. The major products detected include H_2_, CO, formate, ethanol, ethyl acetate, acetaldehyde, and 2‐propanol. The FE_H2_ first decreases as the potential is increased from −0.7 to −0.8 V before slightly increasing upon applying −0.9 V vs RHE. The FE_CO_ shows a reverse trend to that of H_2_ with a maximum at −0.8 V, while overall the FE_CO_ is lower when working in cascade mode compared to only Ag as active electrode, indicating the consumption of the produced intermediate CO on Cu alongside consumption of CO_2_. The FE_HCOOH_ increases with an increased applied reduction potential on the Cu electrode in the cascade mode. This is attributed to the fact that the carbon monoxide reduction reaction (CORR) does not produce any formate on a Cu electrode.^[^ ^29^ ^]^ At lower potentials (−0.7 V vs RHE in this case), CORR is more prominent on the Cu electrode and can outcompete CO_2_RR since CO reduction requires 2 fewer electrons than CO_2_ reduction on a per carbon basis.^[^ ^21^, ^30^ ^]^ The FE_EtOH_ is low at all applied potentials but increases from 2.2% at −0.7 V vs RHE to 6.8% at −0.9 V vs RHE. Notably, we observe significant amounts of 2‐propanol, with its selectivity increasing with more positive applied potential starting from 11% at −0.9 V vs RHE and reaching 32% at −0.7 V vs RHE. This trend matches the findings reported by Kun et al. using supersaturated CO_2_ on an Ag–Cu alloy catalyst.^[^ ^21^ ^]^

**Figure 4:**
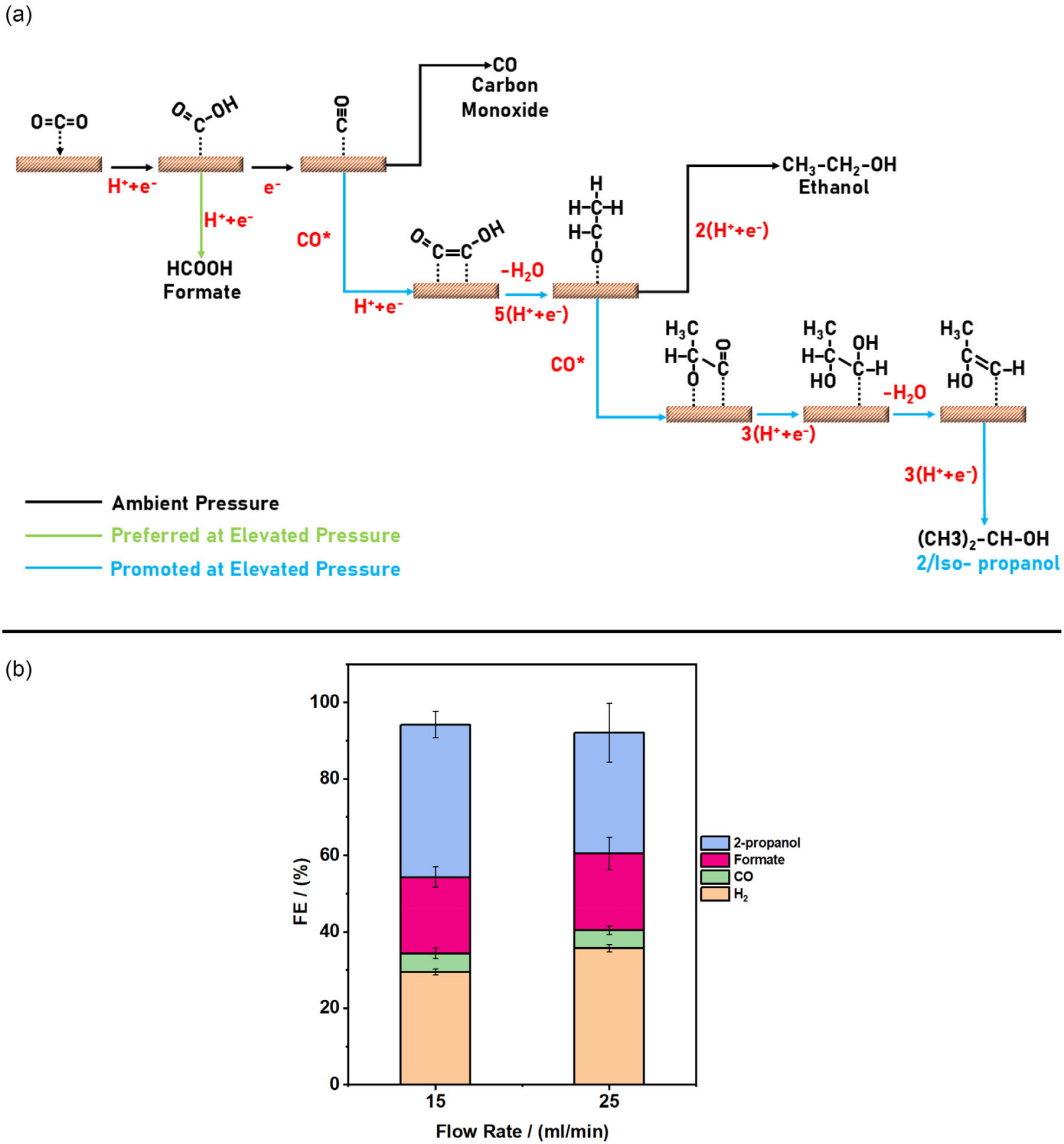
Mechanistic hypothesis and effect of flow rate on 2‐propanol formation. a) Proposed pathway for 2‐propanol formation via CO dimerization and CO‐enol coupling under high *CO surface coverage. b) Effect of electrolyte flow rate (0.1 M KHCO_3_, 25 bar) on 2‐propanol FE in cascade mode (Ag: −1.0 V, Cu: −0.7 V vs RHE). The highest FE for 2‐propanol (≈40%) was observed at 15 mL min^−1^. Error bars represent standard deviation from at least two measurements.

The observed product trends prompted us to consider possible mechanistic pathways, as illustrated in **Figure** **4**a. While the exact pathway towards 2‐propanol formation remains unclear, recent studies have speculated on the responsible reaction mechanism.^[^ ^22^, ^23^ ^]^ In brief, the mechanism includes the dimerization of *CO species through C—C coupling, yielding a reduced dimer (*CO‐COH) species. This can rearrange and further reduce to form a C_2_ enol intermediate (*C_2_H_3_O), a precursor to ethanol. At low potential and high pressures, an increased surface coverage of *CO can promote interactions between the enol and *CO species. Further proton‐coupled electron transfers selectively lead to a C_3_ enol species, culminating in 2‐propanol, resembling the ethanol pathway. The low amounts of ethanol at all tested conditions (especially at higher pressures) suggest that ethanol acts as an intermediate toward 2‐propanol formation under high *CO coverage. In our previous work, we observed an inverse relationship between ethanol and 2‐propanol selectivity under varying pressure and current density conditions.^[^ ^23^ ^]^ This trend is consistent with recent findings of Wang et al. who used isotope‐labeling experiments to confirm that ethanol can serve as a precursor to 2‐propanol via a CO‐assisted coupling pathway.^[^ ^22^ ^]^ In the present study, control experiments performed in cascade mode, where the Ag electrode was deactivated (limiting CO generation), showed a corresponding decrease in 2‐propanol formation and a relative increase in ethanol selectivity. These observations across studies and conditions support the plausibility of an ethanol‐mediated pathway. Future studies using isotopically labeled intermediates or in situ spectroscopy will be necessary to further confirm and resolve the exact mechanistic steps involved.

CO_2_RR using the cascade mode on an Ag—Cu pair at ambient pressure has been shown to create a supersaturated reservoir of CO between the two electrodes.^[^ ^10^ ^]^ It is likely that the combined effect of cascade mode with elevated pressure enables improved solubility and higher surface coverage, enhancing the production of 2‐propanol. To verify this, a control experiment was conducted with the Ag electrode inactive and Cu active at −0.7 V vs RHE (Figure 3b). We find that in this case the FE_2‐propanol_ is only 16.7% while the FE_EtOH_ reaches 16.3%, indicating an inferior selectivity towards 2‐propanol due to lower amounts of surface CO species that are formed directly via CO_2_RR on Cu. Also, the fact that appreciable amounts of ethyl acetate are only observed in the cascade mode indicates higher concentrations of CO on the surface and therefore its enhanced coverage on copper due to a continuous supply by the upstream Ag electrode. Small amounts of ethylene glycol were also detected, with the exact amounts presented in Section 8, Supporting Information. The upper and lower limits of the conversion of CO in the cascade mode (for calculations and discussions see Section 10, Supporting Information) are 88.5% and 85.1% respectively. This underscores the potential of this integrated pressurized cascade catalytic system with simple electrodes to efficiently utilize reactants and intermediates, driving the synthesis of higher‐value reduction products. Strikingly, the C_3_:C_2_ oxygenate ratio is increased in the cascade case reaching a maximum of ≈7 for Ag at −1 V and Cu at −0.7 V vs RHE while for CO_2_RR on Cu only, at the same applied potential, this ratio is only about 0.6 (see Figure S5, Supporting Information).

### 2.3 Effect of Electrolyte Flow Rate

To further enhance the production of 2‐propanol, we probed the effect of flow rate of the CO_2_ saturated electrolyte as it can impact the surface concentration of the intermediates/reactants and their surface coverages.^[^ ^31^, ^32^ ^]^ As shown in Figure 4b, the FE_2‐propanol_ increases from 31.7 to 39.9%, while the FE_H2_ decreases from 35.7 to 29.5% as the flow rate is reduced from 25 to 15 mL min^−1^. The estimated energy efficiency at this condition is calculated as 17.5%, with the calculation details given in section S4. The FE_CO_ decreases slightly from 4.9 to 4.7% with no appreciable changes to formate selectivity. At these moderately high pressures and low current densities, it is noteworthy to mention that the CO_2_RR is not likely affected by the transfer of CO_2_ from the bulk region to the electrode surface.^[^ ^28^ ^]^ However, the flow rate could influence the discharge of CO from the cathode surface to the bulk region. According to Lum et al. a nonequilibrium state is formed on Cu, allowing for a significantly elevated local concentration of CO, surpassing its solubility limit, without diminishing the overall bulk CO_2_ concentration.^[^ ^14^ ^]^ Consequently, a lower flow rate can result in a higher concentration of CO at Cu, thereby increasing the residence time of its subsequent intermediates resulting in an enhanced 2‐propanol selectivity. While these findings suggest a role for local CO concentration in enhancing 2‐propanol formation, a more comprehensive study across a broader range of flow rates would confirm and generalize this trend.

## 3 Conclusion

In conclusion, we have demonstrated the attractiveness of combing elevated pressure with cascade electrocatalysis for converting CO_2_ into C_3_ products. The design of a novel elevated pressure cascade electrochemical cell for CO_2_ electroreduction is presented, using two working electrodes, Ag and Cu. Convective flow is used to transfer CO from Ag to Cu. In this study, at a pressure of 25 bar, tuning the potential and flow rate of the electrolyte led to an FE_2‐propanol_ of about 40% (at −0.7 V vs RHE and 15 mL min^−1^), marking the highest reported selectivity for this uncommon product on a copper electrode. Furthermore, the C_3_:C_2_ oxygenate ratio increased to about 7 in the cascade operation mode compared to a mere 0.6 in the noncascade mode with only Cu as the active electrode. We propose that the synergistic effect of high pressure and cascade operation creates a high concentration of the reaction intermediate CO resulting in its enhanced surface coverage on Cu, thereby promoting the formation of higher alcohols from CO_2_. This strategy highlights the potential of integrating different engineering and operating parameters while using simple electrodes to positively influence the performance of the CO_2_RR process.

While this study focuses on demonstrating selective product formation under elevated pressure in a lab‐scale setup, the insights gained could inform future development of high‐pressure CO_2_ electrolyzers. To enable industrial scalability, future work should explore integration with zero‐gap or flow‐through reactor designs and assess long‐term stability and energy efficiency under practical operating conditions.

## Conflict of Interest

The authors declare no conflict of interests.

## Supporting information

Supplementary Material

## Data Availability

The data that support the findings of this study are available from the corresponding author upon reasonable request.
