# Supplementary material for: Cascade Electrocatalytic Conversion of CO2 to C3 Products at Elevated Pressures
Source: ChemSusChem. 2025 Jun 10;18(15):e202500695. doi: 10.1002/cssc.202500695 (PMC12302312; doi:10.1002/cssc.202500695)
Supplement: Supplementary file 1 — Supplementary Material [file CSSC-18-e202500695-s001.pdf]

Supporting Information

Cascade Electrocatalytic Conversion of CO_2 to C_3 Product at Elevated Pressures

Nandalal Girichandran[1], Lakshmi Mohan[1], Sanne Buisman[1], Andrew Morrison[3,4], Ruud Kortlever[1*]

[1] Process & Energy Department, Faculty of Mechanical Engineering, Large-Scale Energy Storage, Delft University of Technology, Leeghwaterstraat 39, 2628 CB, Delft, The Netherlands

[2] Electrochemical Innovation Lab, University College London, London, WC1E7JE, UK

[3] The Faraday Institution, Didcot, OX11 0RA, UK

Table of Contents

S1. Experimental Information 3
S2. Schematics of the High-Pressure Cascade Setup 5
S3. Gas and Liquid Product Analysis 6
S4. Faradaic and Energy Efficiency Calculation 7
S5. Reactor Assembly Drawing 9
S6. SEM Images of Silver and Copper Foils 10
S7. XRD Spectra of Freshly Prepared Silver and Annealed Copper Oxide Foils 11
S8. Faradaic Efficiencies, Current Densities, and Cell Voltages at 25 bar 12
S9. Ratio of C_3 to C_2 Oxygenates in the Cascade and Non-cascade Modes 13
S10. Calculating CO Conversion in Cascade Mode 14
S11. Comparison With Prior Ag-Cu Cascade Studies and 2-Propanol Synthesis Studies Via Electrochemical CO_2RR 15
S12. A Representative Chronoamperometric Response under Cascade Conditions (25 bar) 16

S1. Experimental Information

Materials

0.1 M potassium bicarbonate ($KHCO_3$, ≥ 99.95% trace metals basis, Sigma Aldrich), and 0.1 M cesium bicarbonate ($CsHCO_3$, 99.9% purity, Sigma Aldrich) were used to prepare the catholyte and anolyte. Silver foil (Ag, 1mm thick, >99.95%, GoodFellow), and copper foil (Cu, 1 mm thick, 99.999% trace metal basis, Sigma Aldrich) served as the two working electrodes, while IrMMO foil was the anode. Nafion 117 (Ion Power GmbH) was cleaned in MilliQ water (18MΩ.cm at 25°C) and used as the ion exchange membrane. HCl (ACS reagent, 37%, Sigma), acetone (Technical Grade, assay ≥ 99%, VWR Chemicals), and H_3PO_4 (85% VLSI, Technic) were used for cleaning and preparation of the electrodes. H_2SO_4 (95-97%, ACS reagent, Honeywell), DMSO (ACS reagent, ≥99.9%, Sigma), phenol (ACS reagent, 99.0-100.5%, Sigma), and D_2O (99.9 atom% D, Sigma) were used for liquid product analysis. An ultrapure water purification system (MilliQ IQ 7000, Merck–Millipore, USA) was used as water source for all experiments. All reagents were used without further purification.

Electrochemical Apparatus and Measurement

The schematic of the entire setup is shown in **S2**. The details of the custom designed cascade reactor housing two working electrodes (Ag and Cu) are given in the results section. A modular clamp system secures the reactor in place and seals the stack leak tight without the need for any nuts and bolts. The catholyte and anolyte are circulated through the reactor using two HPLC pumps (Knauer AZURA P4.1 S with 50 ml pump heads). The outlets from the reactor are connected to external reservoirs through back pressure regulators (Equilibar). The cathode side reservoir is equipped with two inlets – one for the pressurized CO_2 feed and the other coming from the back pressure regulator. An outlet from the reservoir is connected to the pump, while another outlet carries the gaseous stream to an inline GC. In-depth details regarding the assembly can be found in our previous work[1].

Electrochemical experiments were carried out in the custom-designed high-pressure flow setup, using a BioLogic BP300 dual channel potentiostat with EIS analyser. The reactor was operated in a bipotentiostatic mode, offering independent control of the Ag and Cu electrodes against the Ag/AgCl reference electrode (LF 1.6–45 mm, Innovative Instruments Inc., USA). The operation of the two working electrodes and the electrical connections to the external potentiostat were established in a manner similar to that proposed by Gurudayal et al.[2]. Gas products were analysed every 2 minutes using an in-line gas chromatograph (CompactGC 4.0, Global Analyzer Solutions, The Netherlands). Liquid products were collected at the end of the electrochemical experiments and analysed using high-pressure liquid chromatography

(HPLC, Agilent Technologies 1260 Infinity, USA). To cross check and detect intermediate and minor products present in the electrolyte samples 1H-NMR was used (400 MHz Agilent, USA), with the built-in software OpenVnmrJ (University of Oregon, USA). Further details regarding product analysis are provided in section **S3** below.

Electrode Preparation

Ag and Cu foils were cut into pieces of 1.1 cm × 0.6 cm (exposed area in the reactor was 0.6 cm^2). Ag pieces were first mechanically sanded with P1000 and P1200 sandpaper after which the foils were mechanically polished with 3µ and 1µ diamond suspension to a mirror-like finish, devoid of any visible major scratches. After polishing, the Ag foils were sonicated for 5 minutes in 2M HCl and 5 minutes in MilliQ water to remove any impurities from the surface.

Cu foils were mechanically polished with 3 µ and 1µ diamond suspension, following a procedure by Asperti et al.[3], after which they were electropolished in H_3PO_4 for 3 minutes at 2.1V against a carbon rod as a counter and reference electrode. After rinsing with MilliQ water and drying in argon, the Cu foils were annealed in an oven at $230^{O}C$ for 3 hours to oxidize the surface to enhance the roughness and improve its activity under reduction conditions[4].

Electrode Characterization

Scanning Electron Microscopy (SEM, Jeol JSM 6500F) images of the silver and copper oxide electrodes were taken using a Jeol JSM 6500F SEM equipped with an ultradry energy dispersive X-ray spectrometry detector (Thermofisher, USA). to visualize the surface morphology before the experiments. X-Ray diffractograms (XRD) were acquired with a Bruker D8 Advance diffractometer (Bruker, USA) with Bragg-Brentano geometry, employing a graphite monochromator and Vantec position-sensitive detector (Co Kα radiation, divergence slit var12, scatter screen height 8 mm, 40 kV 40 mA).

S2. Schematics of the High-Pressure Cascade Setup

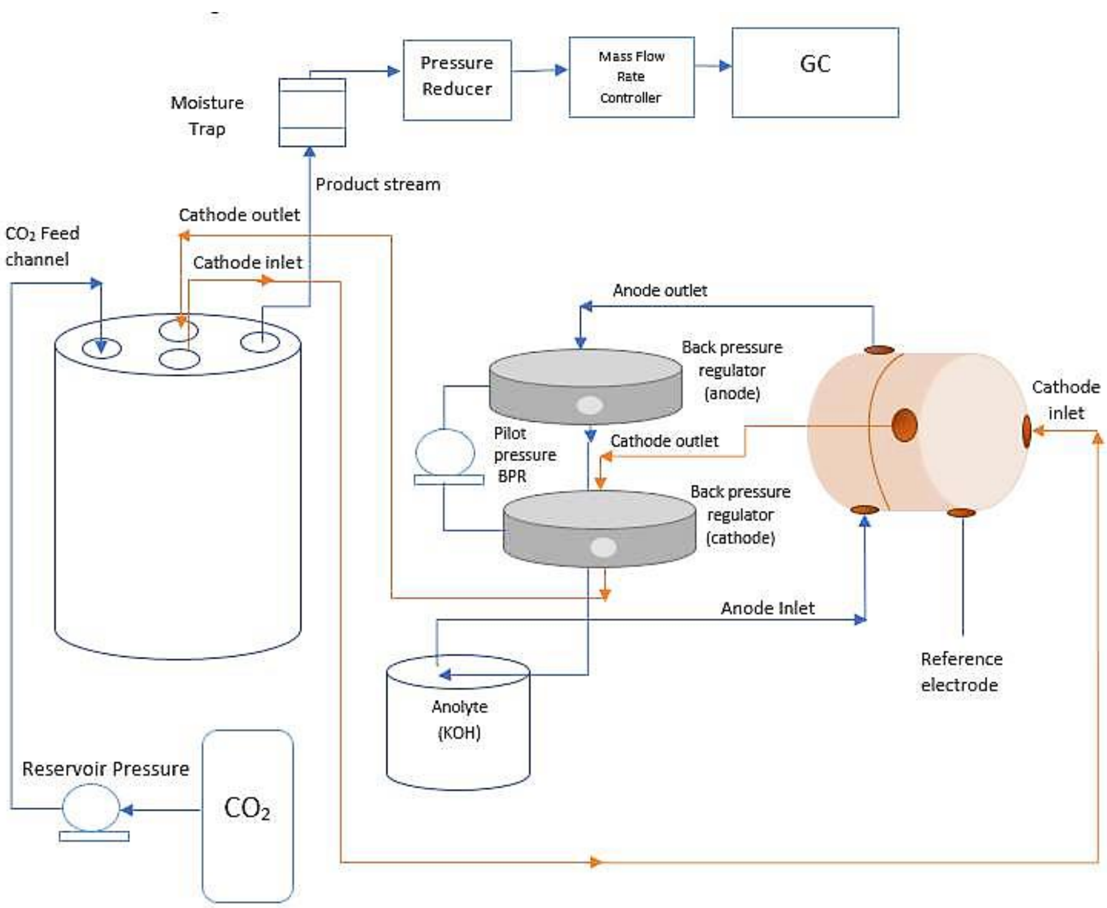

Figure S1. A schematic representation of the high-pressure setup used in this study.

S3. Gas and Liquid Product Analysis

Gas Products - To monitor the gaseous byproducts produced during the reaction, an inline gas chromatograph (CompactGC 4.0, Global Analyzer Solutions, The Netherlands) was utilized. Gas cylinders (Linde Gas Benelux B.V., The Netherlands) containing tailored gas mixtures with CO_2 concentrations ranging from 50 to 8000 ppm were employed for GC calibration. Analysis of gas products was conducted at 2-minute intervals. The GC system features two TCD detectors, one for CO and one for H_2, alongside an FID detector for hydrocarbon analysis (C_1 - C_6). The FID channel is outfitted with an Rtx-1, 5.00 µm (15 m * 0.32 mm) analytical column. The first TCD channel comprises a Carboxen 1010 (3 m * 0.32 mm) pre-column and a Molsieve 5A (5 m * 0.32 mm) analytical column, while the second TCD channel includes a Carboxen 1010 (3 m * 0.32 mm) pre-column and a Molsieve 5A (7 m * 0.32 mm) analytical column, aiding in component separation before detection.

Liquid Products - The liquid products (major product: formate, intermediate products: 2-propanol/iso-propanol, minor products: acetate) obtained post-reaction were analyzed using an Agilent Technologies 1260 Infinity HPLC system from the USA. Standard solutions of desired chemicals (Sigma-Aldrich, USA, >98% formic acid) were prepared for system calibration, with concentrations ranging from 0.1 mM to 50 mM. Each analysis involved injecting 5 µL of the product sample onto two Aminex HPX-87H columns (Biorad) in series, heated to 60°C, with a 1 mM H_2SO_4 solution used as the eluent. Detection of products was carried out using a Refractive Index Detector (RID). For cross-checking and detecting intermediate and minor products present in the electrolyte samples such as ethanol, 1-propanol, 2-propanol, acetaldehyde, ethylene glycol, and propionaldehyde, 1H NMR (400 MHz Agilent, USA), equipped with the built-in software OpenVnmrJ (University of Oregon, USA), was employed. NMR samples were prepared by mixing 630 µL of the catholyte solution with 70 µL of D_2O (99.9 atom % D, Sigma Aldrich, USA), and 30 µL of a freshly prepared mixture containing 50 mM phenol (Sigma Aldrich, USA) and 10 mM DMSO (Sigma Aldrich, USA) as internal standards. The solution was then transferred to 5 mm NMR tubes (Norell Select, USA), which were thoroughly cleaned with acetone using an in-house setup and dried at $80^{O}C$ before sample preparation. Water suppression techniques were employed to obtain clearer spectra.

S4. Faradaic and Energy Efficiency Calculation

We use a gas chromatography (GC) system that directly employs CO_2 as the carrier gas, and for calibration, we use gas mixtures containing ethylene, CO, CH_4, and H_2 at various concentrations, with CO_2 serving as the predominant component. Initially, the high-pressure gas stream from our reservoir passes through a pressure reducer, maintaining a constant pressure of approximately 3 bar, before reaching a mass flow controller (MFC, Bronkhorst). Through the MFC, we regulate the volumetric flow rate to the GC; specifically, we alternate between two values, 0.2 ml_n/min and 8 ml_n/min, to expedite the GC settling time (we call it the switching flow rate technique[1]). The GC columns have been adapted to accommodate CO_2 as the carrier gas, thereby integrating it into the baseline rather than treating it as a distinct entity.

We use the following set of equations to calculate the faradaic efficiency towards products for both the non-cascade mode and cascade mode:

FE gaseous products = (n*F*X*molar flow rate)/ I

where n = number of electrons, F = Faraday's constant (96485 C/mol), X = mole fraction of gaseous product measured by the GC and I = total current applied.

For liquid product analysis,

FE liquid products = (n*F*C*V)/Q_{tot}

, where C = concentration derived from HPLC peak integration, V = volume of the catholyte from which the sample is collected for analysis and Q_{tot} = total charge passed during the experiment. For the cascade mode, the sum of the currents from the two channels is used for the calculation of the FE gases, while the currents from both channels are integrated to calculate the total charge passing through the reactor during the reaction for FE liquids[2].

The energy efficiency (η_{EE})

η_{EE} = ($E_p/E_{applied}$) × FE2-propanol × 100 %

Here:

- E_p is the thermodynamic cell potential, calculated as $E_{ox}-E_{red}$. We use E_{ox} = 1.23V vs SHE and E_{red}=0.1188V vs SHE, leading to E_p= 1.1112V
- $E_{applied}$ = 2.543V, measured for the Cu half-cell in cascade mode
- $FE_{2\text{-propanol}}$=40%

This gives an energy efficiency of approximately 17.5%.

The reduction potential E_{red} is calculated using the Nernst equation to account for elevated CO_2 pressure (25 bar), considering the stoichiometry of 3 CO_2 molecules and 18 electrons in the overall reaction[5]:

$E_{red} = E^0_{red} + (3RT/nF) \times \ln (P_{CO2})$ with $E^0_{red} = 0.105$ V vs SHE

This yields $E_{red} = 0.1188$ V vs SHE.

S5. Reactor Assembly Drawing

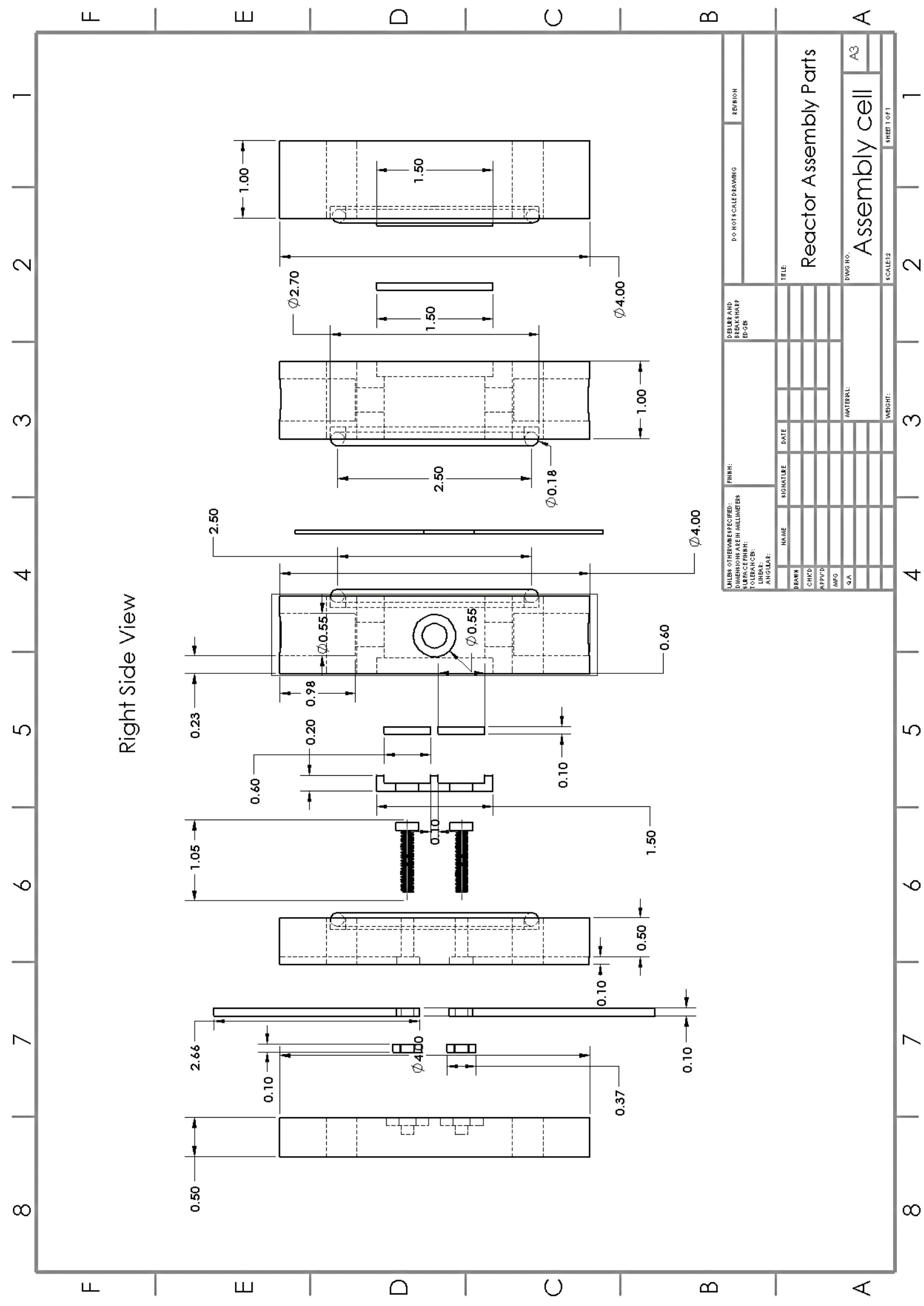

Figure S2. A drawing with measurements for the reactor assembly parts (dimensions in cm).

S6. SEM Images of Silver and Copper Foils

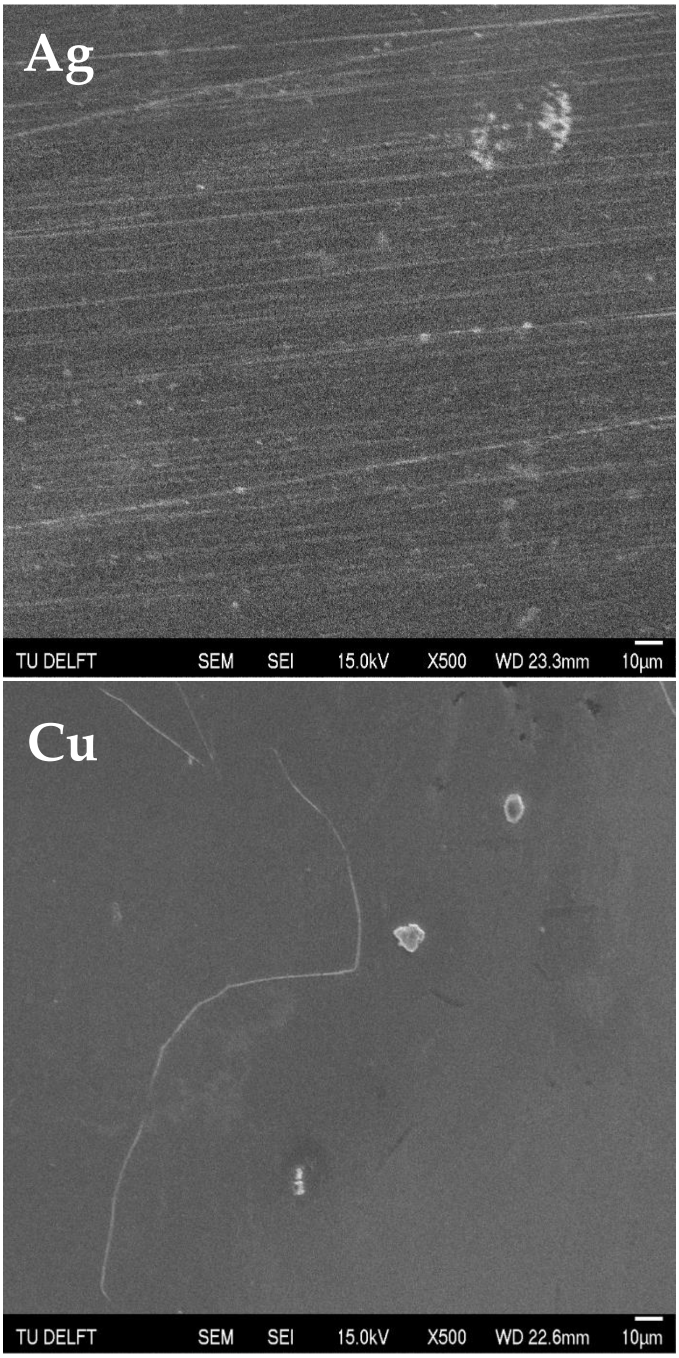

Figure S3. Scanning Electron Microscopy (SEM) images of polished Ag foil and annealed (230 ^{O}C) Cu foil.

S7. XRD Spectra of Freshly Prepared Silver and Annealed Copper Oxide Foils

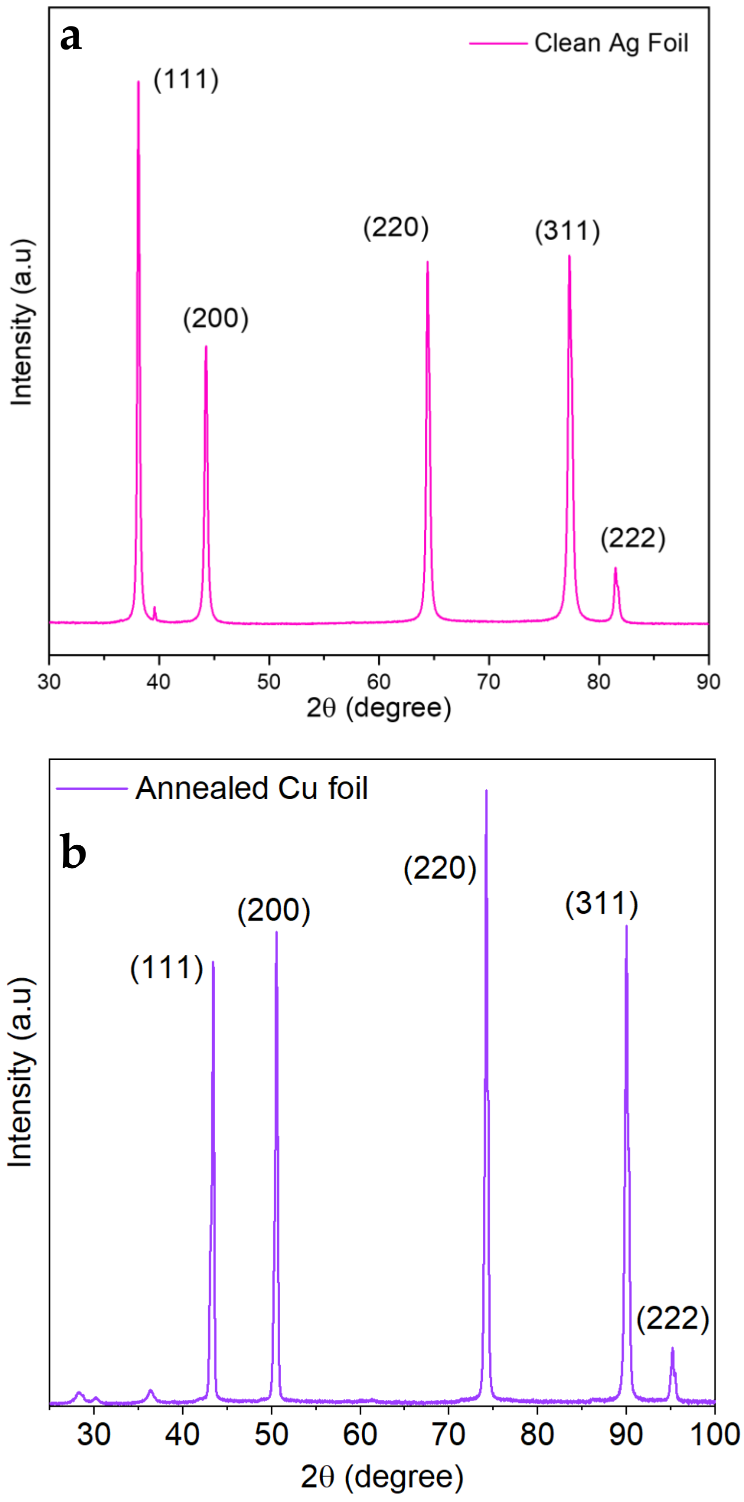

Figure S4. XRD spectra showing the different facets for a) Polished Ag foil, b) Annealed (230 ^{O}C) Cu foil.

S8. Faradaic Efficiencies, Current Densities, and Cell Voltages at 25 bar

Table S1. Faradaic efficiencies of products on bare Ag (-1 V vs RHE) and Cu (-0.7 V vs RHE) electrodes.

Electrode	H_2	CO	Formate	Ethanol	Ethyl Acetate	2-Propanol	Ethylene Glycol	Acetaldehyde	J (mA/cm^2)	Cell Voltage (V)	Total FE (%)
Ag	16.6	53.8	14.5						3.3	2.8	84.9
STD	2.5	2.8	2.2						0.9	0.08	
Cu	29.6	2.6	20.3	16.3		16.9		10.9	2.4	2.4	96.6

Table S2. Faradaic efficiencies of products in cascade mode with the Ag electrode held at -1 V vs RHE.

E_{we} vs RHE (V) applied to Cu	H_2	CO	Formate	Ethanol	Ethyl Acetate	2-Propanol	Ethylene Glycol	Acetaldehyde	J (mA/cm^2) (Ag+Cu)	Cell Voltage (V) (Ag+Cu)	Total FE (%)
-0.7	35.7	4.7	19.7	2.15	2.5	31.5	2.4		4.5	5.5	98.6
	0.9	1.1	3.7	3	1.3	7.8	1.2		0.3	0.02	
-0.8	22.9	16.2	26.6	4.8	4.7	11.6	0.3	3.9	6.5	5.9	90.8
	2.5	11.5	4	6.8	0.4	3.3	0.3	3.9	0.8	0.09	
-0.9	25.6	5.9	33.1	6.8	2.8	10.5	0.4	1.9	6.8	6.1	86.8
	0.6	2.1	3	2.1	2.2	4.4	0.4	1.9	0.1	0.02	

Table S3. Faradaic efficiencies at different flow rates for cascade mode (Ag held at -1 V vs RHE) and Cu oxide held at -0.7 V vs RHE).

Flow Rate (mL/min)	H_2	CO	Formate	Ethanol	Ethyl Acetate	2-Propanol		Ethylene Glycol	Acetaldehyde	J (mA/cm^2)	Cell Voltage (V)	Total FE (%)
15	29.5	4.9	19.9			39.9				4.3	5.4	94.2
	0.8	1.3	2.7			3.5				0.4	0.09	
25	35.7	4.7	19.7	2.2	2.5	31.5		2.4		4.5	5.5	98.6
	0.9	1.1	3.7	3	1.3	7.8		1.2		0.3	0.02	

S9. Ratio of C_3 to C_2 Oxygenates in the Cascade and Non-cascade Modes

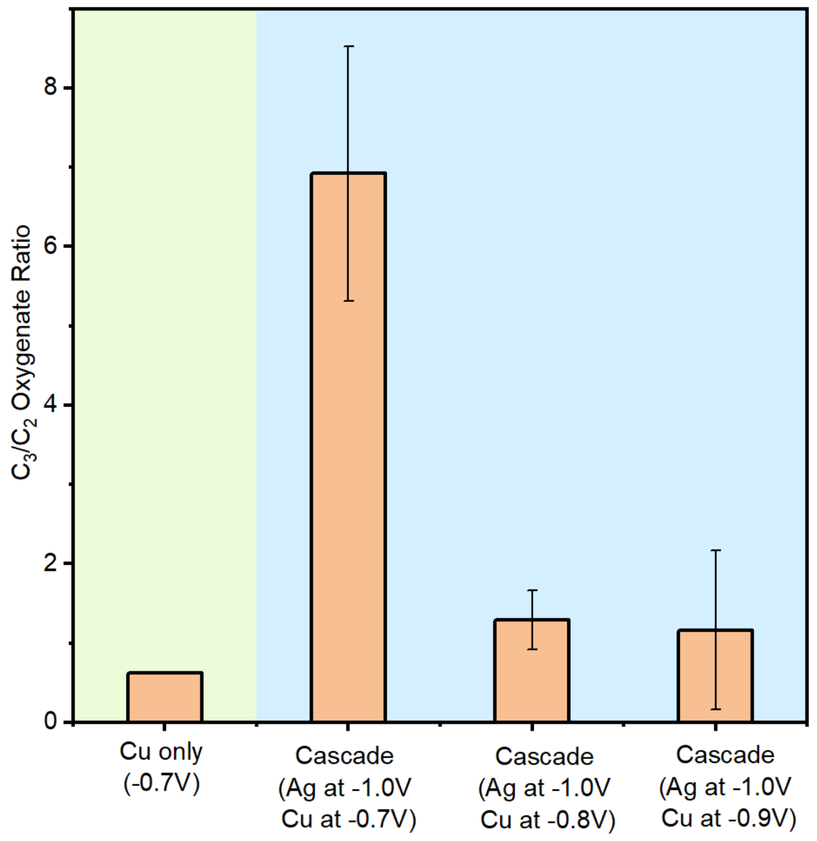

Figure S5. C_3:C_2 Oxygenate ratio for Cu only active mode (at -0.7 V vs RHE), and cascade mode (Ag held at -1 V and Cu at -0.7 V, -0.8 V, and -0.9 V vs RHE).

S10. Calculating CO Conversion in Cascade Mode

C_1-C_2 dimerization during the electrochemical CO_2 reduction reaction results in the formation of C_{2+} products such as 2-propanol. In our high-pressure cascade cell, CO generated at the Ag electrode is transferred to the Cu electrode due to convection, where it adsorbs and reacts to form higher carbon products. Additionally, CO produced at the Cu electrode may desorb, re-adsorb, and undergo further conversion. Since it is challenging to differentiate between these two CO pathways at this stage, we define the CO conversion efficiency by assuming the CO flux (therefore its partial current density) from the Ag electrode during the cascade mode is the same as in the Ag only mode. As suggested by Gurudayal et al., we have considered two limits for this conversion based on the partial current densities of CO formation on copper[2].

The upper limit is defined by assuming that CO formed on Cu in the cascade mode is same as the non-cascade mode with only Cu active.

$$X_{UL,CO} = \left\{ i_{Ag} + i_{Cu} - i_{Cascade} \middle/ i_{Ag} + i_{Cu} \right\} \qquad (1)$$

Where, $X_{UL,CO}$ is the upper limit of CO conversion and i refers to partial current density towards CO and the subscript refers to the electrode/mode of operation.

The lower limit is defined by assuming that no CO is formed on Cu in the cascade mode.

$$X_{LL,CO} = \left\{ i_{Ag} - i_{Cascade} \middle/ i_{Ag} + i_{Cu} \right\} \qquad (2)$$

Where, $X_{LL,CO}$ is the lower limit of CO conversion.

S11. Comparison With Prior Ag-Cu Cascade Studies and 2-Propanol Synthesis Studies Via Electrochemical CO_2RR

Table S4. Comparison to Relevant Studies Reported in Literature.

No	P_{CO2}	Electrolyte	Working Electrode (Cathode)	Cell type	2-Propanol (FE %)	Reference
1	1 bar	0.1M $CsHCO_3$	Sequential Ag-Cu Cascade	Flow Cell	-	[2]
2	1 bar	0.1M $KHCO_3$/ 0.1M KOH	2 Step Ag NC – OD Cu Cascade	Flow Cell	-	[6]
3	1 bar	0.1M $CsHCO_3$	Interdigitated Ag-Cu	Batch Cell	-	[7]
4	10 bar	1M $CsHCO_3$	CO_2-10-$Cu_{94}Ag_6$ Alloy	Autoclave	~ 57	[5]
5*	25 bar	0.5M $KHCO_3$	Polished Cu foam	Flow Cell	~ 11	[8]
6	25 bar	0.1M $KHCO_3$	Sequential Ag-Cu Cascade	Flow Cell	~ 40	This Work

***Our own previous study reporting the first ever observation of 2-propanol on a copper electrode at elevated pressures**

S12. A Representative Chronoamperometric Response under Cascade Conditions (25 bar)

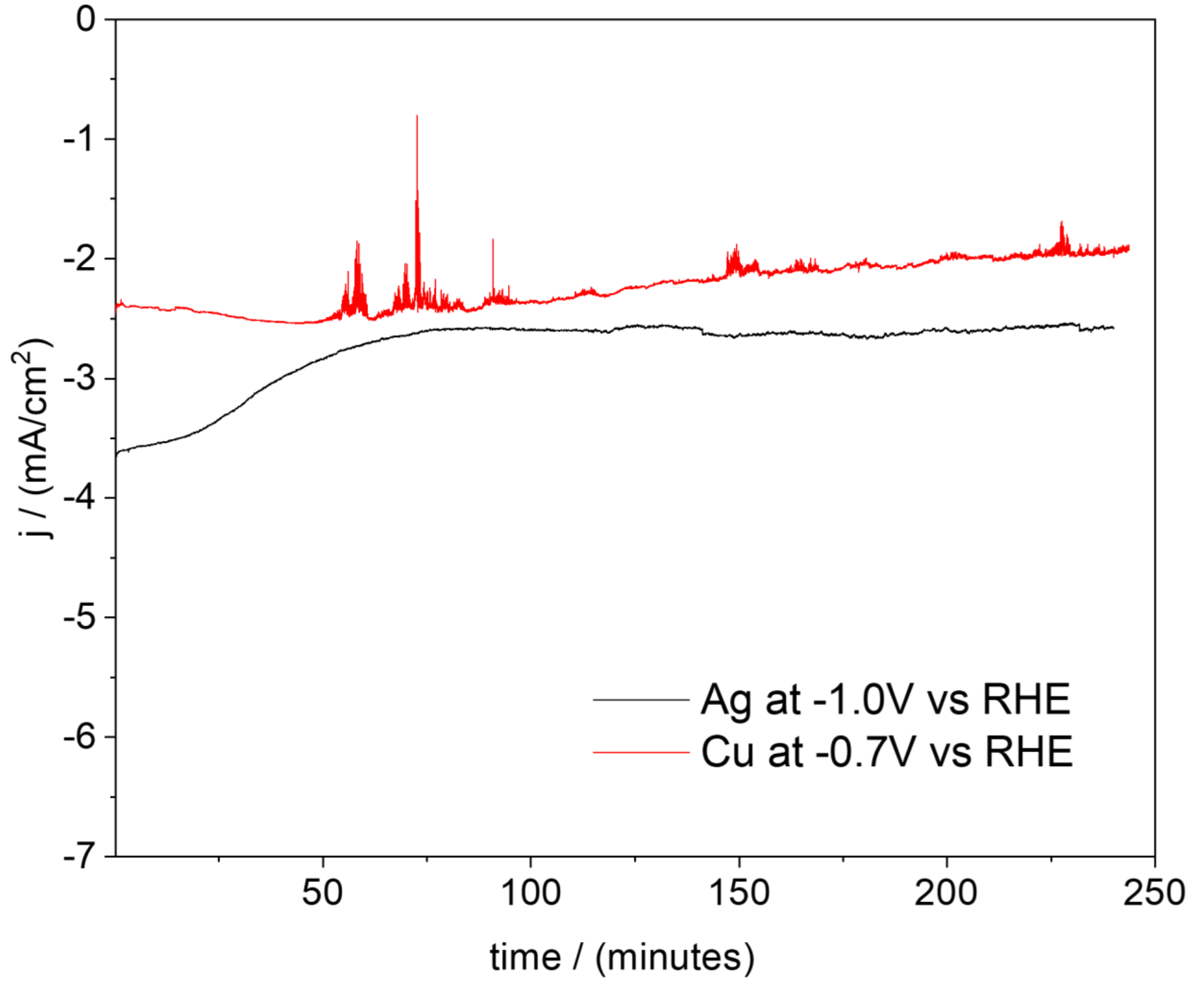

Figure S6. Chronoamperometric response of the cascade system at 25 bar CO_2 pressure with Ag held at –1.0 V and Cu at –0.7 V vs RHE in 0.1 M $KHCO_3$. The experiment was conducted for about 4 hours, showing sustained current and stable product distribution throughout the duration.

References

[1] A. R. T. Morrison, N. Girichandran, Q. Wols, R. Kortlever, *Journal of Applied Electrochemistry* **2023**, *1*, 1-10.

[2] G. Gurudayal, D. Perone, S. Malani, Y. Lum, S. Haussener, J. W. Ager, *ACS Applied Energy Materials* **2019**, *2*, 4551-4559.

[3] S. Asperti, R. Hendrikx, Y. Gonzalez-Garcia, R. Kortlever, *ChemCatChem* **2022**, *14*, e202200540.

[4] L. Diao, Y. Liu, F. Chen, H. Pan, D. P. d. Lara, H. Liu, Y. Cheng, F. Luo, *Materials Reports: Energy* **2023**, *3*, 1-8.

[5] K. Qi, Y. Zhang, N. Onofrio, E. Petit, X. Cui, J. Ma, J. Fan, H. Wu, W. Wang, J. Li, J. Liu, Y. Zhang, Y. Wang, G. Jia, J. Wu, L. Lajaunie, C. Salameh, D. Voiry, *Nature Catalysis* **2023**.

[6] N. Theaker, J. M. Strain, B. Kumar, J. P. Brian, S. Kumari, J. M. Spurgeon, *Electrochimica Acta* **2018**, *274*, 1-8.

[7] Y. Lum, J. W. Ager, *Energy and Environmental Science* **2018**, *11*, 2935-2944.

[8] N. Girichandran, S. Saedy, R. Kortlever, *Chemical Engineering Journal* **2024**, *487*.
